# The impact of land structure and function mixing on carbon emission efficiency: an examination of 268 Chinese cities

**DOI:** 10.3389/fpubh.2026.1893717

**Published:** 2026-07-07

**Authors:** Qixuan Li, Luwei Liao, Zhenxiang Chen, Shuxuan He

**Affiliations:** 1 School of Economics and Management, Hunan University of Technology, Zhuzhou, China; 2 School of Management, Wuhan Technology and Business University, Wuhan, China; 3 School of Economics and Trade, Changsha, China

**Keywords:** carbon abatement policy, carbon emission efficiency, Chinese cities, information entropy, mixed land use

## Abstract

Extreme heat and disease transmission triggered by global warming pose severe threats to human health, creating an urgent need to promote low-carbon development. It is widely recognized that mixed land-use strategies play a critical role in reducing carbon emissions and mitigating global warming. In contrast to existing literature that solely focuses on either land structure mix or land function mix, this research integrates both into a unified analytical framework for comparative analysis. This approach aims to clarify the key directions that mixed land-use strategies should prioritize. By employing the information entropy method, this study utilizes remote sensing imagery and Point of Interest (POI) data to characterize structural and functional mixed land use across 268 prefecture-level cities in China, and further analyzes their impacts on carbon emission intensity. The experimental results show that land structure mix significantly reduces the carbon emission intensity; in contrast, the land function mix increases the carbon emission intensity. The mechanism analysis shows that the former generates positive externalities by promoting economic agglomeration, offsetting the negative effects of traffic congestion, whereas the latter intensifies the urban traffic pressure and does not show significant agglomeration effects. The spatial econometrics results further show that a significant spatial spillover effect of functional mixing, which further amplifies the carbon emission pressure in the city. The article further proposes policy recommendations.

## 1 Introduction

Greenhouse gas (GHG) emissions from human activities have raised the global average temperature by about 1.43 °C above the pre-industrial level, and 2024 became the first year to exceed the 1.5 °C threshold (1). The Sixth Assessment Report of the Intergovernmental Panel on Climate Change (IPCC) clearly states that climate change has already caused significant negative impacts on global public health: extreme heat increases premature deaths in all regions, the risks of climate-sensitive diseases and malnutrition continue to rise, and threats to mental health are also intensifying (2). In this context, reducing carbon emissions and curbing global warming have become urgent tasks for protecting human health. Land use change is the second largest source of global carbon emissions after fossil fuel combustion. According to the IPCC Sixth Assessment Report, agriculture, forestry, and other land uses account for 13–21% of total anthropogenic GHG emissions (3). However, current land-use carbon reduction measures often require a trade-off between economic gains and ecological responsibilities: restricting urban expansion to protect forests and carbon sinks may constrain local economic growth, while prioritizing high-intensity development may further exacerbate carbon emissions. This dilemma highlights the urgent need for a land use pattern that can reconcile ecological benefits with economic viability. Mixed land use, which integrates multiple functions within a single spatial unit, has emerged as a potential solution to this trade off by optimizing resource allocation and reducing systemic inefficiencies.

In recent years, mixed land use has gained increasing attention and has gradually extended into two core dimensions. Land structure mixing focuses on the area proportions and combination relationships of different land use types, while land function mixing focuses on the spatial proximity and interaction of different socio-economic activities. Together, these two dimensions constitute the full connotation of mixed land use, and focusing on only one of them cannot fully capture its actual impact on urban operations. Improper levels of structural and functional mixing may increase transport emissions and energy consumption, thereby amplifying the threat of climate change to public health. Therefore, to maximize the co-benefits of climate mitigation and public health, identifying and clarifying the differentiated effects of land structure mixing and land function mixing on carbon emission efficiency has become an increasingly urgent research direction.

Existing research on the relationship between mixed land use and carbon emissions has roughly formed three strands. The first strand focuses on structural mixing based on land use types, the second on functional mixing based on functional diversity, and the third on comprehensive assessments that combine the two. These studies have used quantitative tools such as information entropy, proximity indicators, and comprehensive measurement models, providing important theoretical and empirical foundations for understanding the mechanisms through which mixed land use affects carbon emissions.

The first strand of research examines the impact of land structure mixing on carbon emissions, taking the proportional diversity of land use types as the entry point and commonly using information entropy to measure the degree of mixing across various land use types. For example, Xu et al. (4) used information entropy and the Tapio decoupling model to analyze the decoupling relationship between land structure mixing and carbon emissions in the Hohhot-Baotou-Ordos-Yulin urban agglomeration. Kay et al. (5) assessed the carbon sink potential of agroforestry systems in Europe and found that combining trees and crops can effectively improve carbon sequestration efficiency. The advantage of such studies is their wide data coverage and long time series, enabling them to reveal the statistical relationship between land type proportions and carbon emissions. However, their limitation is that they focus only on area proportions and cannot reflect the interactions between different functions within the same space.

The second strand examines the impact of land function mixing on carbon emissions, taking the spatial proximity of socio-economic activities as the entry point and analyzing how functional mixing (e.g., jobs-housing balance, facility accessibility) affects residents’ travel and carbon emissions. For instance, Gong et al. (6) investigated the effect of land use on household travel carbon emissions, and Chai et al. (7) proposed low-carbon optimization strategies based on the carbon emission mechanisms of daily household travel. The strength of these studies is that they are close to residents’ actual behavior and can reveal the mechanisms of functional mixing at the micro level. However, their limitations include a heavy reliance on questionnaire data with limited coverage and insufficient discussion of the negative effects that may arise from excessive agglomeration of functional mixing.

The third strand calls for incorporating both structural and functional mixing into a unified framework. Song et al. (8) systematically compared multiple measures of mixed land use, distinguishing between entropy-based methods (based on proportional diversity) and accessibility-based methods (based on spatial proximity). Shi et al. (9) pointed out that existing studies often overlook functional compatibility as a crucial characteristic, resulting in incomplete evidence. Zhou et al. (10) and Tang et al. (11) theoretically argued for the unity of the two dimensions. Zheng et al. (12) proposed a systematic framework that integrates the two types of methods. The advantage of these studies is their comprehensive perspective, providing a conceptual basis for subsequent research. Nevertheless, their limitation is that most of them remain at the theoretical or review level; empirical studies that truly place both dimensions in the same analytical framework for systematic comparison are still scarce, and tests of their differentiated mechanisms and spatial spillover effects are even rarer.

Therefore, the information entropy method adopted in this study measures the effects of both land structure mixing and land function mixing on carbon emission efficiency within a unified framework, offering notable advantages in dimensional integration, mechanism identification, and empirical testing. First, information entropy can simultaneously capture the number of land use types and the evenness of their area distribution, effectively revealing the orderly evolution of land use structures in urbanized areas (13). Applying it to land cover classification data and urban POI data enables parallel quantification of structural and functional mixing, compensating for the lack of framework integration in previous studies. Second, traditional methods mostly focus on the overall effect of mixed land use and find it difficult to distinguish the differentiated mechanisms of structure and function. In contrast, information entropy separately constructs a structural index and a functional index, making it possible to independently examine their different impacts on carbon emission efficiency, providing a new empirical path to explain the controversial “beneficial vs. detrimental” findings. Third, unlike the third-strand studies that mainly remain at the conceptual framework level, the information entropy method relies on publicly available land use raster data and urban POI data, enabling large-scale, long-term quantitative measurement across the full sample of cities. This provides a reproducible and extensible empirical foundation for testing causal relationships between mixed land use and carbon emission efficiency.

China, as the world’s largest energy consumer, has contributed about 12% (±2%) of global warming (14), and its urbanization process has extensively adopted mixed land use strategies. In contrast to the low-density, suburban development patterns of European and American countries, Chinese cities are typically characterized by high density and mixed functions. Meanwhile, as the world’s second most populous country, China has many cities where population density far exceeds that of comparable cities globally, underscoring an urgent need to examine the relationship between global warming and population health, and makes Chinese cities ideal samples for studying the link between mixed land use and carbon emissions, as well as for providing empirical evidence for climate-health co-governance.

The remainder of this paper is structured as follows. Section 2 reviews relevant theories and proposes research hypotheses; Section 3 briefly introduces the data and methodology; Section 4 presents benchmark regression and robustness tests; Section 5 discusses the mechanism of action and spatial spillover effects respectively, and the discussion and conclusions are presented in Section 6.

## 2 Research hypothesis

The concept of mixed land use, which entails shifts in production, lifestyle, and energy use, is viewed as a vital strategy for addressing urban challenges such as inefficient land use and traffic congestion (15). It is regarded as beneficial for reducing carbon emissions. For example, combining residential, commercial, and public facilities within urban areas can reduce commuting distances and times, thereby increasing efficiency. Such practices also decrease resource and energy consumption. Moreover, mixed land use fosters innovation and knowledge exchange by bringing together diverse industries, institutions, and talents, which enhances innovation rates and production efficiency, further reducing carbon emissions (16, 17). However, some scholars argue that mixed land use might increase traffic flows and congestion, and impede land use specialization, leading to segmented labor markets and lower productivity (18). Additionally, excessive mixing is argued to dilute employment density and disperse resources (19). These divergent views may arise from the conflation of land use structures and functions—for example, knowledge exchange relates to mixed functions, whereas labor specialization relates to land structure. Nonetheless, the link between mixed land use and reduced carbon dioxide emissions is clear. Thus, this study hypothesizes that:

Mixed land use has a significant impact on urban carbon emissions, but the directions of influence differ between land structure mixing and land function mixing (Hypothesis 1).

Mixed land use, by bringing together diverse activities within a compact spatial unit, can generate significant agglomeration economies. The literature has delineated three classical sources of agglomeration externalities: input sharing, labor market pooling, and knowledge spillovers (20). First, the spatial concentration of residential, commercial, and industrial activities facilitates the shared use of infrastructure and public services, thereby reducing per-unit production costs and fixed investment burdens. Second, the co-location of diverse industries creates a thick labor market where workers can efficiently match with employers, reducing job search frictions and unemployment risk. Third, compact spatial arrangements promote face-to-face interactions, which are particularly essential for the transmission of tacit knowledge (21). These positive agglomeration externalities, in turn, contribute to carbon emission reduction through multiple channels: they lower pollution control costs per unit of output, enhance labor productivity and thus reduce energy intensity per unit of GDP, and accelerate technological innovation that improves overall production efficiency (22–27).

However, agglomeration also generates negative externalities. The concentration of diverse activities within limited geographic space inevitably increases local travel demand and may exacerbate traffic congestion, which in turn raises carbon emissions through increased vehicle idling, frequent stops, and overall energy consumption (28, 29). Moreover, the relationship between mixed land use and travel behavior is not unequivocally positive: some studies have found that mixed land use may increase traffic flows and congestion, impede land use specialization, and lead to fragmented labor markets and lower productivity (17, 18). This implies that whether mixed land use ultimately reduces or increases carbon emissions depends on the relative strength of positive versus negative agglomeration externalities. When positive externalities (such as economies of scale and knowledge spillovers) dominate, mixed land use may lower carbon emissions; when negative externalities (such as traffic congestion) prevail, mixed land use may instead raise carbon emissions. Based on this reasoning, this study proposes the following hypothesis:

The impact of mixed land use on carbon emissions originates from the trade-off between positive and negative agglomeration externalities, with land structure mixing and land function mixing exhibiting different directions of influence (Hypothesis 2).

## 3 Methods and data

### 3.1 Methods

With the advancement of mixed land use practices, corresponding research has flourished. There are three primary quantitative methods to assess mixed land use. The first method evaluates land use diversity, using entropy indices based on the proportional areas or functions of various land use types (8). The second method examines land use proximity, assessing the accessibility among complementary infrastructure, public services, and land types. The third method combines the previous two into a holistic evaluation system (12).

Currently, there are two main approaches to measuring the degree of land use mix: entropy-based methods, which capture land use diversity through the proportional distribution of different land use areas or functional counts (8); accessibility-based methods, which measure the degree of functional mixing by evaluating the spatial proximity among complementary infrastructure, public services, and land use types (30).

In contrast, information entropy has three major advantages that make it particularly suitable for our research design. First, it simultaneously captures the number of land use categories and the evenness of their spatial distribution. As discussed in our literature review, information entropy can effectively interpret the orderly and rational evolution of land use patterns (13, 31). This property is equally valuable for measuring functional mixing using POI data: a city where POIs are evenly distributed across different activity types (e.g., catering, services, enterprises, etc.) receives a higher entropy score, thus providing a more accurate measure of true functional diversity rather than being dominated by a single category. Second, information entropy relies on publicly available land use raster data and urban POI data, which are available for all 268 cities over the entire study period, making large-scale, long-term panel analysis feasible. Third, the entropy measure produces a standardized index (bounded between 0 and 1) for both structural and functional mixing, allowing direct comparison of their effect sizes—a key requirement for answering our core research question of which dimension should be prioritized in low-carbon planning.

Given that information entropy can reflect the number of land use types and the even distribution of land areas for each type, and can effectively explain the ordered and rational evolution of land use structures in urbanized areas (13), this paper selects this method. Therefore, this paper selects this method to reflect the degree of mixed land use. Equation 1 calculates the structural mixed land use index based on information entropy. The calculation formula is as follows:


$$\mathrm{Mix}=-\sum_{i=1}^{n}[(\mathrm{bi}/m)\ln(\mathrm{bi}/m)]$$ (1)

In this context, Mix represents the information entropy of land use, ranging from 0 to 1, where values closer to 1 indicate greater land use diversity. For measuring land structure diversity, n is the number of land use types, bi is the area of a specific land use type, and m is the total area of all land use types within an urban area. Considering the relationship between land use and carbon emissions, this paper divides land types into nine categories, namely, farmland, forest, shrub, grassland, water, glacier, bare land, construction land and wetland. For assessing land function diversity, the study utilizes Point of Interest (POI) information entropy, with Amap urban POI data serving as the foundation. Here, n denotes the number of POI types, bi is the count of a specific POI type, and m is the total count of POI types in the city, reflecting urban functional diversity. Considering the relationship between socio-economic activities and carbon emissions, this paper selects POI data of nine categories, namely catering services, accommodation services, scenic spots, companies and enterprises, shopping services, finance and insurance, science, education and culture, medical care and health, and government agencies for analysis. Building on the previous step, this study constructs a benchmark regression model aligned with Hypothesis 1, as detailed in Equation 2.


$$\mathrm{PGC}E_{\mathrm{it}}=\alpha_{0}+\alpha_{1}{\mathrm{Mix}}_{\mathrm{it}}+\alpha_{2}\text{Control}s_{\mathrm{it}}+\delta_{i}+\varepsilon_{\mathrm{it}}$$ (2)

In the model, $\mathrm{PGC}E_{\mathrm{it}}$ represents the carbon emission intensity for the i city in the t year, measured by carbon dioxide emissions per unit of GDP, where lower values indicate lower carbon emission intensity and thus higher efficiency. ${\mathrm{Mix}}_{\mathrm{it}}\;$ captures the mixed-use land conditions, including both land structure mix (Mixstructure) and land function mix (Mixfunction). Control variables are introduced to account for various factors: Urb represents the urbanization rate; Urban scale (Scale) uses the city’s total population; Economic level (Rgdp) is gauged by per capita GDP, with its squared term assessing the Environmental Kuznets Curve hypothesis; The industrial structure (Is) is measured by the proportion of the secondary industry output value to the GDP in each city; Technological level (Tech) is measured by the ratio of urban technological expenditure to GDP, aligning with the theory that higher R&D investment boosts production and environmental efficiency; Foreign direct investment openness (Fdi) is considered through actual foreign capital investment, reflecting the “pollution transfer” effect; Environmental regulation (Eri) employs pollution control investment as a proxy, examining its effect on carbon efficiency in line with the “Porter hypothesis” (32, 33). This study adopts the approach suggested by scholars like (42), utilizing pollution control investment as a metric for environmental regulation. This method allows for an evaluation of environmental regulation’s impact on carbon emission efficiency, ensuring a comprehensive analysis of its effects. The level of public transportation (Bus) in a city is represented by the total volume of public bus passenger transport. To minimize heteroscedasticity effects, logarithmic transformations are applied to non-structured variables in the regression analysis.

### 3.2 Data sources

This study utilized data from 268 Chinese prefecture-level cities, including four directly-administered municipalities, as the primary data collection region. We collected and analyzed land-use data and Point of Interest (POI) data from 2012 to 2019. The annual data collection date is set as the last day of the year. To account for the significant impact of home isolation and suspended industrial activities during the COVID-19 pandemic on carbon emissions, the data collection period for this study concluded at the end of 2019.

The land-use data used in this study were sourced from the research conducted by Yang and Huang (34). This dataset encompasses various categories such as farmland, forest, shrubbery, grassland, water bodies, glaciers, bare land, construction land, and wetlands, with a spatial resolution of 30 m. The POI data were obtained from Amap and cover eleven categories related to daily life and economic activities. These categories include accommodation services, government institutions and social organizations, healthcare, sports and leisure services, business residences, science, education, and cultural services, financial and insurance services, shopping services, companies and enterprises, dining services, and scenic spots. Since the Chinese government has not yet officially released city-level carbon dioxide emission data, we follow the common practice in the academic literature by summing the carbon emissions from urban natural gas, liquefied petroleum gas (LPG), electricity, heat, and transportation to obtain total urban carbon emissions, and then calculate carbon emission intensity on this basis. Specifically, China’s power system is divided into six regional grids: North China, Northeast China, East China, Central China, Northwest China, and Southern China. Each grid has its own baseline emission factor. Using these emission factors together with city-level electricity consumption data, we calculate the carbon emissions from electricity use for each city. For transportation carbon emissions, we combine energy consumption data from the China Statistical Yearbook with passenger and freight volumes from the China City Statistical Yearbook to estimate city-level transportation emissions. For heat consumption, given that district heating mainly uses coal-fired boilers, we adopt a thermal efficiency value of 70%. Based on the calorific value of raw coal and the heating demand, we determine the required amount of raw coal and convert it into standard coal equivalent. Following the emission coefficients provided in the IPCC 2006 Guidelines (35), we calculate the carbon emissions generated from district heating. Below, we provide descriptive statistics for the key variables used in our analysis (see Table 1).

**Table 1:** Descriptive statistics of variables.

Variables	N	Means	SD	Min	Max
PGCE	2,144	1.241	0.791	−1.999	4.298
Mixstructure	2,144	0.507	0.135	0.115	0.777
Mixfunction	2,144	0.822	0.0710	0.626	0.979
Urb	2,144	0.523	0.166	0.120	1
lnScale	2,144	4.730	0.784	2.715	7.816
lnRgdp	2,144	0.434	0.537	−2.155	2.744
Is	2,144	0.468	0.114	0.118	0.837
Tech	2,144	0.00300	0.00300	0	0.0500
Fdi	2,144	0.0170	0.0180	0	0.205
Eri	2,144	0.644	0.140	0.198	0.945
lnBus	2,144	9.186	1.230	4.605	13.15

## 4 Results

### 4.1 Spatial characteristics

**Figure 1:**
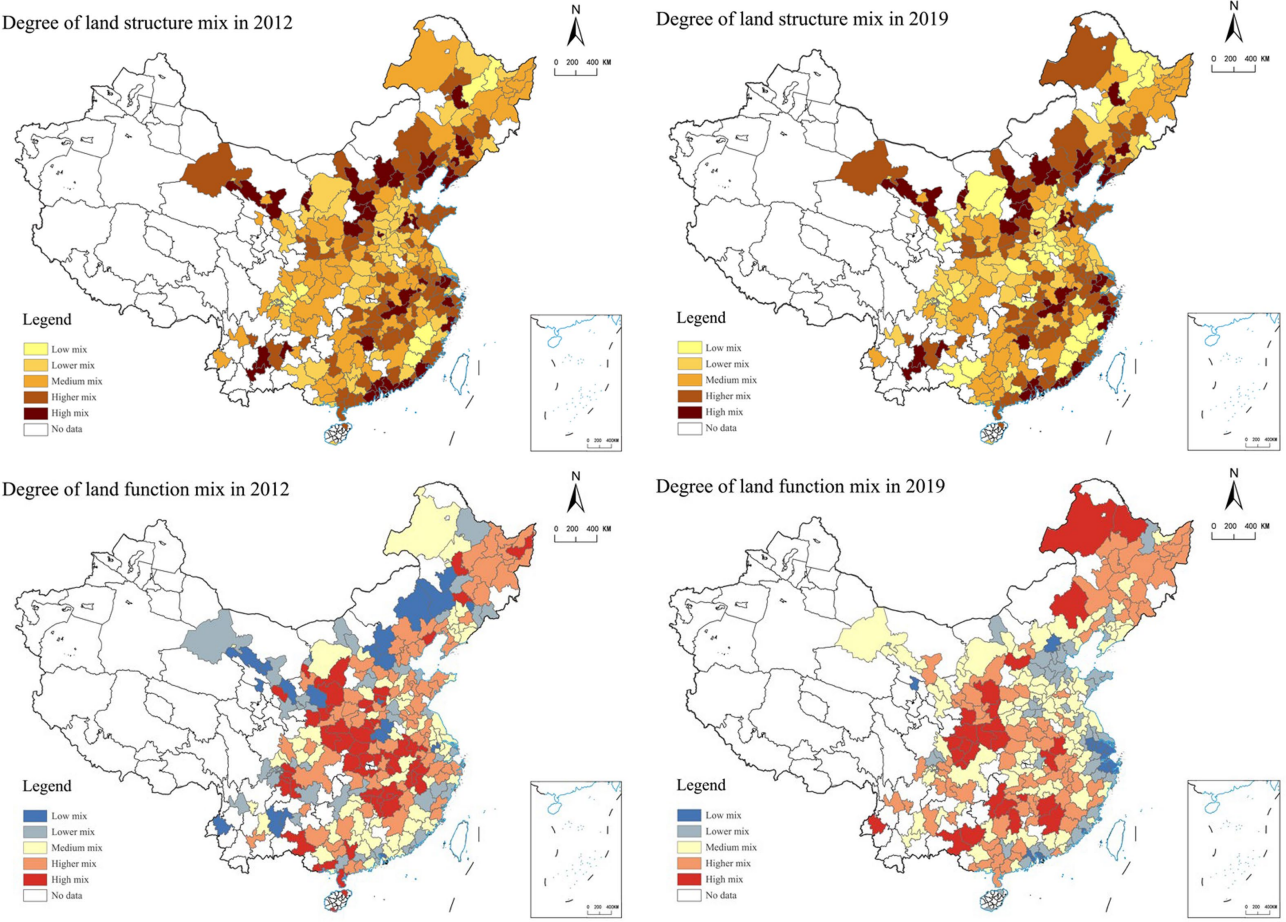
Spatial distribution of mixed land use in 2012 and 2019.

First, the natural breaks method was used to classify land structural mix and function mix into five levels: low mix, lower mix, medium mix, higher mix, and high mix. These classifications were then visualized using ArcGIS to obtain the spatial evolution of land mixed use in 268 Chinese cities for the years 2012 and 2019 (Figure 1).

As can be seen from the figure, in terms of land structural mixing, regions with relatively high mixing degrees are relatively stable. Whether in 2012 or 2019, they are mainly concentrated in national-level urban agglomerations such as the Beijing-Tianjin-Hebei region, the Guangdong-Hong Kong-Macao Greater Bay Area, and the Yangtze River Delta urban agglomeration. The core cities within the urban agglomerations have the highest mixing degree, while the surrounding cities have slightly lower mixing degrees. However, regions with lower mixing degrees have changed significantly. In 2012, there were fewer regions with low mixing degrees, but in 2019, some underdeveloped regions showed low mixing degrees. One possible reason for this phenomenon is that underdeveloped regions often promote the agglomeration of a single industry (such as manufacturing parks) through the “low-price land supply + policy preferential” model to attract investment, leading to convergence in land use types.

In terms of land functional mixing, compared with 2012, the areas with low mixing decreased significantly in 2019. This phenomenon benefits from the rise of models such as “commercial-office-residential mixed land use” and “industrial communities.” It is worth noting that the land functional mixing degree in the eastern coastal areas has always been low. Possible reasons include the formation of specialized industrial clusters in the eastern coastal areas (such as Wenzhou’s footwear industry, Yiwu’s small commodities, and Suzhou’s electronic manufacturing), leading to a high degree of specialization in land functions. In addition, the eastern coastal areas are mostly mountainous and hilly, and the plain areas are divided by rivers and bays, which is also one of the important reasons.

### 4.2 Benchmark regression

In Table 2, the regression results in columns (1) and (2) respectively describe the relationships between land structure mix, land function mix, and carbon emission intensity with control variables. The results show that the relationship between land structure mix and carbon emission intensity is not significant, while land function mix increases carbon emission intensity.

**Table 2:** Benchmark regression results.

Variables	(1)	(2)	(3)	(4)	(5)	(6)
	PGCE	PGCE	TSLS	TSLS	logCO	logCO
Mixstructure	0.444 (1.387)		−85.149*** (27.899)		−74.280*** (24.369)	0.589 (1.472)
Mixfunction		1.044*** (0.246)		7.662*** (1.578)	0.978** (0.461)	7.715*** (1.565)
Urb	0.800*** (0.195)	0.832*** (0.196)	0.400 (0.316)	1.048*** (0.225)	0.483* (0.286)	1.052*** (0.225)
lnScale	−0.136** (0.062)	−0.128** (0.062)	−0.013 (0.121)	−0.080 (0.066)	−0.022 (0.110)	−0.080 (0.066)
lnRgdp	−0.169*** (0.051)	−0.168*** (0.050)	0.123 (0.148)	−0.165*** (0.054)	0.086 (0.131)	−0.167*** (0.055)
Is	−0.581** (0.229)	−0.515** (0.225)	−0.470 (0.439)	−0.095 (0.257)	−0.422 (0.395)	−0.093 (0.257)
Tech	6.443 (5.146)	8.217 (5.477)	12.391 (9.516)	19.273** (8.174)	13.269 (9.101)	19.320** (8.176)
Fdi	1.556** (0.742)	1.520** (0.734)	7.030*** (2.596)	1.116 (0.791)	6.275*** (2.303)	1.075 (0.790)
Eri	−0.227** (0.114)	−0.259** (0.113)	−0.532** (0.245)	−0.448*** (0.131)	−0.521** (0.220)	−0.448*** (0.132)
lnBus	0.081*** (0.030)	0.078*** (0.029)	0.099** (0.046)	0.055** (0.028)	0.093** (0.041)	0.055* (0.028)
Constant	0.940 (0.774)	0.267 (0.446)	36.127*** (11.638)	−6.401*** (1.506)	30.698*** (10.170)	−6.695*** (1.543)
Coefficient of first stage			0.119*** (0.038)	−1.322*** (0.217)		
DWH *p* value			0.0013	0.0000		
*F*			11.556	69.169		
*N*	2,144	2,144	2,144	2,144	2,144	2,144
*R* ^2^	0.832	0.834	0.271	0.762	0.406	0.761

*, **, and *** denote significance levels of 10, 5, and 1%, respectively.

These results are apparently contrary to our hypothesis that improving land use can reduce carbon emissions, which may be due to endogeneity issues. Given the theoretical concern of endogeneity between land mixed-use and carbon emission efficiency, the Ordinary Least Squares (OLS) estimation results may be biased. For example, reverse causality may occur: local governments in areas with suboptimal carbon emission efficiency might intensify efforts to adjust land use and industrial structures, directly affecting mixed land use. Additionally, the model incorporates numerous control variables to mitigate omitted variable biases but cannot fully ensure all influences on urban land mixing are accounted for. To address this issue, we conducted Hausman tests separately for land structure mix and land function mix. The results, which reject the null hypothesis at a 1% significance level, indicate that the explanatory variables are indeed endogenous, suggesting that land mixed-use is an endogenous variable. Thus, we employ the Two-Stage Least Squares (TSLS) method to obtain more accurate estimates of the net effects of land mixed-use on carbon emission intensity.

The key to TSLS lies in selecting appropriate instrumental variables, and in urban environment research, terrain relief is widely adopted for this purpose. It satisfies the exogeneity condition because terrain is a naturally formed factor that does not directly affect carbon emission intensity. Meanwhile, it meets the relevance condition as rugged terrain is generally avoided for residential or industrial construction in urban development but remains viable for planting, thereby influencing mixed land use. The analysis results of TSLS are presented in Columns (3) and (4) of Table 2, from which it can be observed that the *p*-values obtained from the Durbin–Wu–Hausman (DWH) test consistently remain below 0.05 across all models, confirming that land mixed-use is indeed endogenous to carbon emission efficiency. This underscores the necessity of applying the TSLS method for model estimation. Additionally, the first-stage *F*-statistics for each model exceed the critical value specified by Stock and Yogo (36) at the 20% level of bias, indicating the absence of weak instrument issues in the models and thus satisfying the fundamental requirements of TSLS.

Results in Column 3 indicate a negative correlation between land structure mix and carbon emission intensity: for every one-unit increase in the former, the latter decreases by 85.149%. In contrast, land functional mix shows a positive correlation with carbon emission intensity: each additional unit of the former leads to a 7.662% increase in the latter. In other words, land structure mix helps reduce carbon emissions, while functional mix increases them.

Results in Columns (5) and (6) of Table 2 present the TSLS estimates with both land structure mix and land function mix included simultaneously. Compared with the separate regressions in Columns (3) and (4), after controlling for land function mix, the coefficient of land structure mix decreases from −85.149 to −74.280, indicating that land function mixing itself has a positive effect on carbon emissions and thus partially offsets the emission-reduction effect of structural mixing. Conversely, after controlling for land structure mix, the coefficient of land function mix increases from 7.662 to 7.715, indicating that land structure mixing itself has a negative effect on carbon emissions and thus partially suppresses the emission-increasing effect of functional mixing. The signs and significance of both coefficients remain unchanged, confirming the robustness of our core conclusion that land structure mixing reduces carbon emission intensity while land function mixing increases it.

### 4.3 Robustness test

To enhance the reliability of the conclusions, this paper conducts robustness tests from two aspects: changing sample data and adjusting model estimation strategies. First, the samples of municipalities directly under the central government are excluded. As provincial-level administrative units directly administered by the Chinese government, municipalities typically have larger built-up areas and larger resident populations, and their capabilities and logics in political and economic management decision-making differ from those of ordinary cities. Eliminating their influence can verify the robustness of the conclusions. Columns (1) and (2) of Table 3 present the experimental results: for each unit increase in land structure mixing degree and functional mixing degree, carbon emission intensity decreases by 79.615% and increases by 7.603%, respectively. This result is close to the benchmark regression results, indicating that the core conclusions of this paper have strong robustness.

**Table 3:** Robustness test results of changing sample data.

Variables	(1) PGCE	(2) PGCE	(3) PGCE	(4) PGCE	(5) PGCE	(6) PGCE
Mixstructure	−79.615*** (25.117)		−78.530*** (24.060)		−76.809** (30.647)	
Mixfunction		7.603*** (1.589)		7.654*** (1.581)		20.937* (12.307)
Control variables	Yes	Yes	Yes	Yes	Yes	Yes
Coefficient of first stage	0.126*** (0.038)	−1.318*** (0.218)	0.133*** (0.039)	−1.362*** (0.224)	0.150** (0.064)	−0.550* (0.341)
DWH *p* value	0.0007	0.0000	0.0005	0.0000	0.0204	0.0708
*F*	12.723	70.558	14.170	70.976	9.304	5.802
*N*	2,112	2,112	2,112	2,112	1,296	1,296

*, **, and *** denote significance levels of 10, 5, and 1%, respectively.

Second, since the municipal districts of some Chinese cities (e.g., Chongqing, Kunming, and Zhengzhou) are not contiguous with their main urban areas but are instead located outside local counties or county-level cities, their carbon emission intensity may vary accordingly. Therefore, such enclave-type municipal districts are excluded for robustness testing. Additionally, considering that Dongguan City has no municipal districts, resulting in significant differences in administrative divisions compared to other sample cities, it is also excluded. The results are presented in Columns (3) and (4) of Table 3, where the coefficients and directions of the land structure mixing index and functional mixing index are consistent with the benchmark regression, highlighting the robustness of the experiment.

Third, in recognition of substantial disparities in carbon emission efficiency among resource-oriented cities primarily engaged in natural resource extraction, such as minerals and forests, this study, in line with the ‘National Sustainable Development Plan for Resource-Oriented Cities (2013–2020),’ excluded resource-oriented cities in the Two-Stage Least Squares (TSLS) regression for robustness testing [Column (5) and (6)]. The results indicate that although there are slight differences in coefficient values compared to the benchmark regression, the core conclusions remain intact, confirming the reliability of the experimental findings.

Fourth, the number of urban post offices in 1984 is used as an instrumental variable for robustness testing, where the number of post offices in 1984 reflects the commercial and population concentration of the region at that time, and historically, areas with concentrated commerce and population were more likely to form mixed land-use patterns, satisfying the relevance condition, while it has no direct impact on current carbon emissions, meeting the exogeneity condition. The test results are presented in Columns (1) and (2) of Table 4. After replacing the instrumental variable, the experimental findings remain consistent with the benchmark regression results, indicating that the core conclusions of this paper exhibit strong robustness.

**Table 4:** Robustness test results of adjusting model estimation strategies.

Variables	(1) PGCE	(2) PGCE	(3) PGCE	(4) PGCE	(5) PGCE	(6) PGCE
Mixstructure	−60.235** (30.392)		−70.676*** (21.603)		−85.149*** (26.817)	
Mixfunction		11.603** (5.227)		6.817*** (1.266)		7.662*** (1.377)
Control variables	Yes	Yes	Yes	Yes	Yes	Yes
Coefficient of first stage	−0.002** (0.001)	0.012** (0.005)	0.137*** (0.040)	−1.421*** (0.146)		
DWH *p* value	0.0128	0.0126	0.0005	0.0000	0.0003	0.0000
*F*	6.385	8.281	16.990	110.506	11.556	69.169
*N*	2,144	2,144	1876	1876	2,144	2,144

*, **, and *** denote significance levels of 10, 5, and 1%, respectively.

Besides, when investigating the impact of land use change on carbon emissions, it is essential to account for temporal lag effects. These effects arise due to inertia and response dynamics within the land use system. After a change in land use, a certain amount of time is required for both ecological and socio-economic systems to adapt to the new conditions. Consequently, there exists a temporal mismatch between land use changes and carbon emission efficiency. This study addresses this by incorporating a one-period lag of variables to eliminate the influence of this temporal factor. As depicted in Columns (3) and (4) of the results, the experimental results remain consistent with the benchmark regression, indicating high robustness.

Finally, the TSLS estimation method was replaced with GMM. Compared to TSLS, GMM is more efficient when instrumental variables are not fully exogenous. The results are presented in Columns (5) and (6) of Table 4, where the coefficient for land structure mix is negative and that for land functional mix is positive, consistent with the baseline regression. Overall, a series of robustness tests indicate that the core conclusions of this paper are highly robust.

## 5 Further analysis

**Figure 2:**
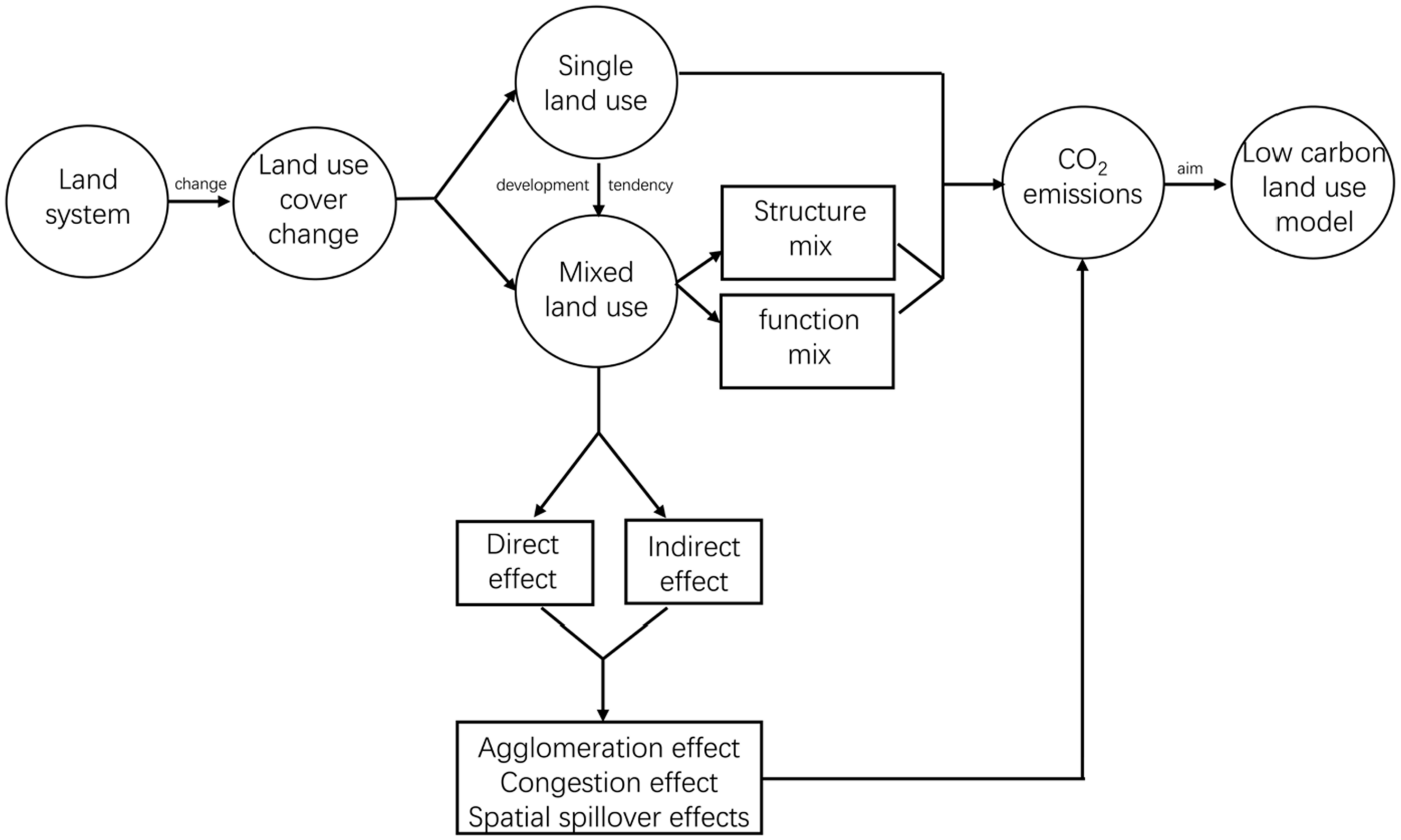
The pathway of mixed land use influencing carbon emissions.

The previous section has theoretically elaborated and analyzed the mechanisms influencing carbon emission efficiency under the context of mixed land use (as described in Figure 2), laying the foundation for empirical testing.

In line with Hypothesis 2, this study aims to investigate whether land mixed-use contributes to the enhancement of economic agglomeration. In our research, economic agglomeration pertains to the degree of concentration within local land areas. To measure this, we must consider the varying sizes of administrative areas in different cities and their relative positions on a national scale. Therefore, we have chosen to employ the geographic concentration index as our method for assessing the level of economic agglomeration. Equation 3 is constructed to quantify the urban economic agglomeration degree. The employed approach is as follows:


$$\mathrm{EC}O_{\mathrm{it}}=\frac{\mathrm{ec}o_{it}∕\sum_{i=1}^{N}\mathrm{ec}o_{\mathrm{it}}}{A_{it}∕\sum_{i=1}^{N}A_{\mathrm{it}}}$$ (3)

In the equation, ECO represents the level of economic agglomeration, where a higher value indicates a greater degree of economic agglomeration. ‘eco’ represents urban employment, ‘*i*’ signifies city *i*, ‘*t*’ represents the year, ‘*N*’ is the number of cities, and ‘*A*’ denotes the administrative area of each city. Notably, the instrumental variable ‘terrain’ in the baseline regression fails to meet the exogeneity requirement. Thus, we adopt ‘the number of cultural heritage sites’ as the instrumental variable for mechanism testing. On the one hand, cultural heritage sites represent a single land use type and are typically incompatible with mixed land use, satisfying the relevance condition. On the other hand, cultural heritage sites do not directly influence economic agglomeration, thereby satisfying the exogeneity condition.

The results presented in Columns (1) and (2) of Table 5 indicate that land structure mix contributes to increased economic agglomeration, with each unit increase in the former leading to a 15.673% rise in the latter. In contrast, the correlation between land function mix and economic agglomeration is not significant. This finding provides a sound explanation for the observation in the baseline regression that land structure mix reduces carbon emission intensity, which can be largely attributed to its role in promoting economic agglomeration.

**Table 5:** Mechanism analysis results.

Variables	(1) ECO	(2) ECO	(3) TJ	(4) TJ
Mixstructure	15.673** (7.524)		0.188*** (0.062)	
Mixfunction		67.725 (599.611)		0.744*** (0.145)
Control variables	Yes	Yes	Yes	Yes
Constant			1.792*** (0.085)	1.234*** (0.153)
Coefficient of first stage	−0.004** (0.001)	−0.001 (0.008)		
DWH *p* value	0.0074	0.9099		
*F*	3.095	0.008		
*N*	2,144	2,144		
*R* ^2^			0.490	0.508

*, **, and *** denote significance levels of 10, 5, and 1%, respectively.

However, the current results have not yet clarified the mechanism underlying the increase in carbon emission intensity caused by land function mix. As Hypothesis 2 suggests, mixed land use may have a certain impact on transportation efficiency. A certain degree of land mixing may promote public transportation choices, improve transportation efficiency, and reduce carbon emission intensity. However, excessive mixing may also lead to congestion, thereby increasing carbon emission intensity. To test the above hypothesis, this paper employs the ‘traffic jam index (TJ)’ from the ‘China Major Cities Traffic Analysis Report (2015–2019)’, jointly released by Amap, the Ministry of Transport, and Alibaba Cloud, as the indicator for urban congestion. Defined as the ratio of the average actual travel time of urban residents for a single trip to the travel time under free-flow conditions, this index can reflect urban congestion to a certain extent. The specific dataset covers 45 cities in 2015, 58 cities in 2016, 95 cities in 2017, 48 cities in 2018, and 48 cities in 2019, based on which empirical tests are conducted.

The results in Columns (3) and (4) of Table 5 reveal the relationship between mixed land use and traffic congestion, showing that both land structure mixing and land function mixing contribute to congestion to a certain extent, with the latter having a higher coefficient. Combined with previous conclusions, it can be inferred that both land structure mixing and function mixing increase congestion, thereby raising carbon emission intensity. However, land structure mixing promotes economic agglomeration, which in turn reduces carbon emission intensity, and the reduction effect is greater than the increase in carbon emission intensity caused by congestion; thus, land structure mixing overall decreases carbon emission intensity. In contrast, land function mixing fails to obtain the positive externalities of economic agglomeration while still increasing congestion, resulting in an overall increase in carbon emission intensity.

In summary, the impact of mixed land use on carbon emissions originates from the trade-off between positive and negative agglomeration externalities. This is consistent with recent findings by Zhang et al. (37), who demonstrated that the emission-reduction effect of economic agglomeration is particularly pronounced in large cities and economically developed regions—where positive agglomeration externalities are more likely to dominate. This supports our conclusion that when positive externalities prevail, mixed land use tends to reduce carbon emissions, whereas when negative externalities (such as congestion) dominate, it may instead increase emissions.

Furthermore, as a regional rather than local issue, carbon dioxide emissions cannot be contained by administrative boundaries, leading to significant spatial dependence among neighboring cities in this regard (38). To verify this effect, this paper constructs a spatial econometric model using a geographic distance-based spatial weight matrix, which fully accounts for spatial dependence between adjacent cities (Equation 4). A key feature of this model is spatial lag, encompassing lags in the dependent variable, independent variables, and error terms. Different spatial econometric models are constructed using various combinations of these lags.


$$\;\;\mathrm{PGC}E_{\mathrm{it}}=\text{ρWPGC}E_{\mathrm{it}}+\alpha l_{n}+\mathrm{\beta Mi}x_{\mathrm{it}}+\text{θWMi}x_{\mathrm{it}}+\varepsilon$$ (4)

Where $\;\mathrm{PGC}E_{\mathrm{it}}$ denotes the carbon emission intensity of city i in year t , $\mathrm{Mi}x_{\mathrm{it}}$ represents the mixed land use of city i in year t , W is an n*n spatial nesting weight matrix, $l_{n}$ is an *n* × 1 unit matrix, β is the coefficient associated with mixed land use, ρ and θ are spatial autocorrelation coefficients, and ε is the random error term, assumed to follow a normal distribution; when ρ ≠ 0, θ = 0, and ε = 0, the model conforms to the Spatial Lag Model (SAR), which incorporates a spatial lag operator for the dependent variable, implying that all independent variables affect other regions through spatial transmission mechanisms; when *ρ* = 0, *θ* = 0, and ε ≠ 0, the model conforms to the Spatial Error Model (SEM), which assumes that interactions among regions are realized through the error term, with spatial spillover effects driven by random shocks; and when *ρ* ≠ 0, *θ* ≠ 0, and ε = 0, the model conforms to the Spatial Durbin Model (SDM), which includes spatial lag operators for both the dependent and independent variables, allowing for the simultaneous analysis of spatial spillover effects of both the dependent and independent variables across regions.

The premise of the spatial econometric model is that variables are spatially autocorrelated. Therefore, before conducting spatial econometric regression, it is first necessary to test whether the data has spatial autocorrelation. The global Moran’s *I* was selected to calculate the spatial autocorrelation of the explained variable (Table 6). It can be seen that the explained variable is significantly positive at the 1% level, indicating that carbon emission intensity has spatial autocorrelation, and further spatial econometric analysis can be carried out.

**Table 6:** Spatial autocorrelation test results.

Year	2012	2013	2014	2015	2016	2017	2018	2019
Moran’s	0.072***	0.075***	0.090***	0.114***	0.117***	0.067***	0.066***	0.077***

Table 7 presents the results of the spatial econometric analysis. Columns (1) to (3) report the estimation outcomes of the SAR, SEM, and SDM models, respectively. Based on the principle of maximizing the Log-likelihood value, the SDM model was selected for interpretation. As shown in the table, land structure mix did not exhibit a significant spatial spillover effect, whereas land function mix had a statistically significant spatial spillover effect on carbon emission intensity. Combined with the previous empirical findings, this implies that the promotion effect of land function mix on carbon emission intensity has a spatial spillover effect. Although spatial econometric models can estimate the coefficients of the spatial lag terms of explanatory and explained variables, the magnitude of these coefficients does not truly reflect their actual impact on the explained variable. To address this issue, partial derivatives were used to derive the real effects of explanatory variables on the explained variable [Columns (4) to (6) in Table 7]. The results indicate that a one-unit increase in land function mixing leads to a 1.086% increase in carbon emission intensity in the local city through spatial spillover effects, while it has no significant impact on carbon emission intensity in other cities.

**Table 7:** Spatial econometric analysis results.

Variables	(1)	(2)	(3)	(4)	(5)	(6)
	SAR	SEM	SDM	Direct effect	Indirect effect	Total effect
Mixstructure	0.368 (1.009)	0.309 (1.018)	−0.061 (1.041)			
Mixfunction	1.000*** (0.218)	1.039*** (0.224)	1.076*** (0.253)	1.086*** (0.257)	−0.097 (3.038)	0.989 (2.931)
Control variables	Yes	Yes	Yes			
*N*	2,144	2,144	2,144			
*R* ^2^	0.107	0.066	0.131			
Log-likelihood	−612.0303	−612.3318	−605.2509			

*, **, and ***denote significance levels of 10, 5, and 1%, respectively; log-likelihood and *R*^2^ are the coefficients of the land function mix experiment model; the coefficient of the real effect comes from the SDM of land function mix.

## 6 Discussion and conclusion

Global warming poses multiple threats to human health, with extreme heat, the spread of infectious diseases, disaster impacts, and the burden of chronic diseases continually exacerbating public health risks. Faced with this complex situation, medicine and public health have focused on technology-oriented approaches such as disease treatment and behavioral intervention, while climate science and environmental engineering have devoted themselves to developing emission reduction technologies and adaptation strategies. However, the dimensions of health impacts are complex and multifaceted, covering physiological, psychological, accidental injury, and chronic disease aspects. Moreover, attributing specific health outcomes to climate change involves considerable uncertainty, making direct empirical testing with health data challenging. Therefore, this study prioritizes investigating the effects of land structure mixing and land function mixing on carbon emission efficiency, aiming to provide an intermediate empirical foundation for subsequent assessments of the health consequences of land use.

Comparison with existing literature. Compared with the block-scale study of Tuffour et al. (39, 40), this study focuses on the entire urban area, and the travel behavior examined is different. The travel behavior at the block scale in Tuffour et al. (39, 40) is, to some extent, a kind of internal traffic efficiency, whereas the travel behavior at the city scale is regional mobility—shaped by land function mixing—such as job-housing commuting and commercial activities. Therefore, our findings only appear to conflict with theirs, but in fact they do not. Meanwhile, the studies of Xu et al. (4) and Choo et al. (41) also support our conclusion, as they consider mixed land use as a “double-edged sword,” whose impact on emissions largely depends on land-use conditions and city types.

The main conclusions of this study are as follows. Land structure mixing significantly reduces carbon emission intensity, whereas land function mixing increases it. Land structure mixing generates positive externalities by promoting economic agglomeration, and this positive effect outweighs the negative effect caused by traffic congestion; land function mixing fails to produce a significant agglomeration effect and instead aggravates urban traffic pressure. Moreover, land function mixing exhibits a notable spatial spillover effect, further amplifying local carbon emission pressure. Based on these findings, this paper proposes the following policy recommendations. First, spatial planning should distinguish between the different effects of structural mixing and functional mixing. It is advisable to encourage integrated allocation of land for residential, industrial, and commercial uses, while controlling excessive concentration of high-intensity functions such as education, healthcare, and administration in local areas to avoid inducing commuting tides and traffic congestion that would weaken low-carbon benefits. Second, given that functional mixing tends to trigger congestion, mixed-use development should be integrated with rail transit stations and public transport hubs, i.e., the transit-oriented development (TOD) model. By improving slow-traffic systems, optimizing feeder services, and implementing congestion pricing, reliance on private cars can be reduced, the share of green travel increased, and the risk of carbon emission rebound from functional intensification effectively mitigated. Third, at the stage of urban master planning, comprehensive consideration should be given to the synergistic optimization of land structure mixing and land function mixing. Polycentric development strategies should be emphasized to counteract the potential congestion-enhancing effects of functional mixing, with due attention to the heterogeneity of city sizes. At the urban renewal level, a certain degree of functional mixing should be achieved to enhance regional vitality, while excessive functional mixing that may lead to imbalances in the spatial distribution of functional vitality should be avoided. The structural-functional mixing index proposed in this study can serve as a practical tool to guide land use allocation and evaluate the low-carbon performance of redevelopment projects. These conclusions provide theoretical and empirical support for mitigating the threat of global warming to human health.

This study has two limitations. First, the data coverage is limited in time, only extending to 2019 and not covering the years after the COVID-19 pandemic. The spread of remote work and changes in travel patterns may have structurally affected the relationship between mixed land use and carbon emissions; future research should use longer-term post-pandemic data to test the robustness of our findings. Second, direct health data verification is lacking. This study does not include health outcome variables in the empirical model. Future research can incorporate public health data to provide more direct evidence for the link between land use and population health.

## Data Availability

The original contributions presented in the study are included in the article/supplementary material, further inquiries can be directed to the corresponding author.
